# Supplementary material for: Elevated Expression of Cell Adhesion, Metabolic, and Mucus Secretion Gene Clusters Associated with Tumorigenesis, Metastasis, and Poor Survival in Pancreatic Ductal Adenocarcinoma
Source: Cancers (Basel). 2024 Dec 3;16(23):4049. doi: 10.3390/cancers16234049 (PMC11640259; doi:10.3390/cancers16234049)
Supplement: Supplementary file 1 [file cancers-16-04049-s001.zip › Supplementary Figures.pdf]

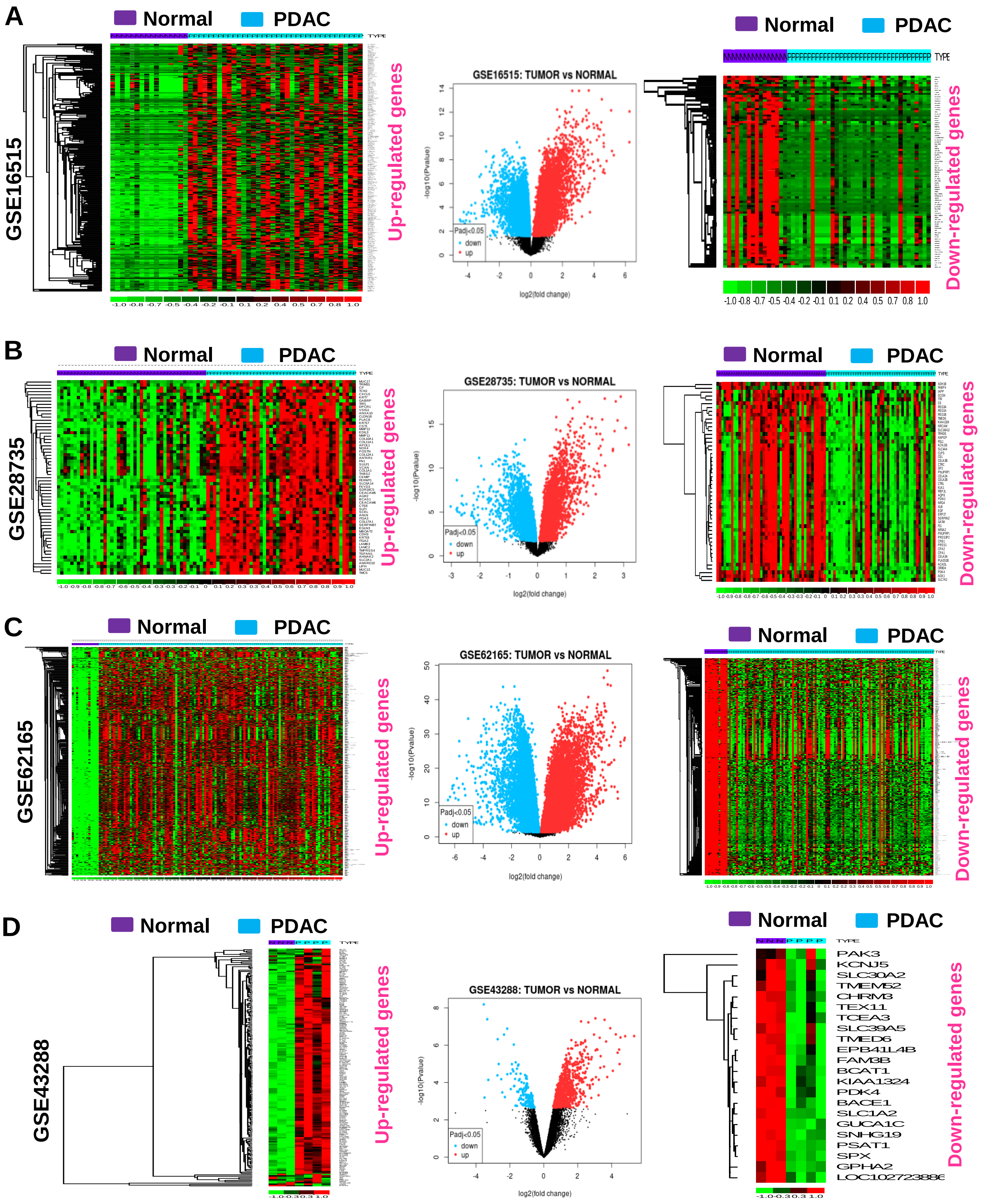


**Figure S1.** Pancreatic adenocarcinoma mRNA expression profiles GSE16515, GSE28735, GSE62165, and GSE43288 were utilized to analyze the differentially expressed genes. **A-D**) The heat maps show the expression pattern of differentially dysregulated genes and the volcano plot of DEGs in their profiles.

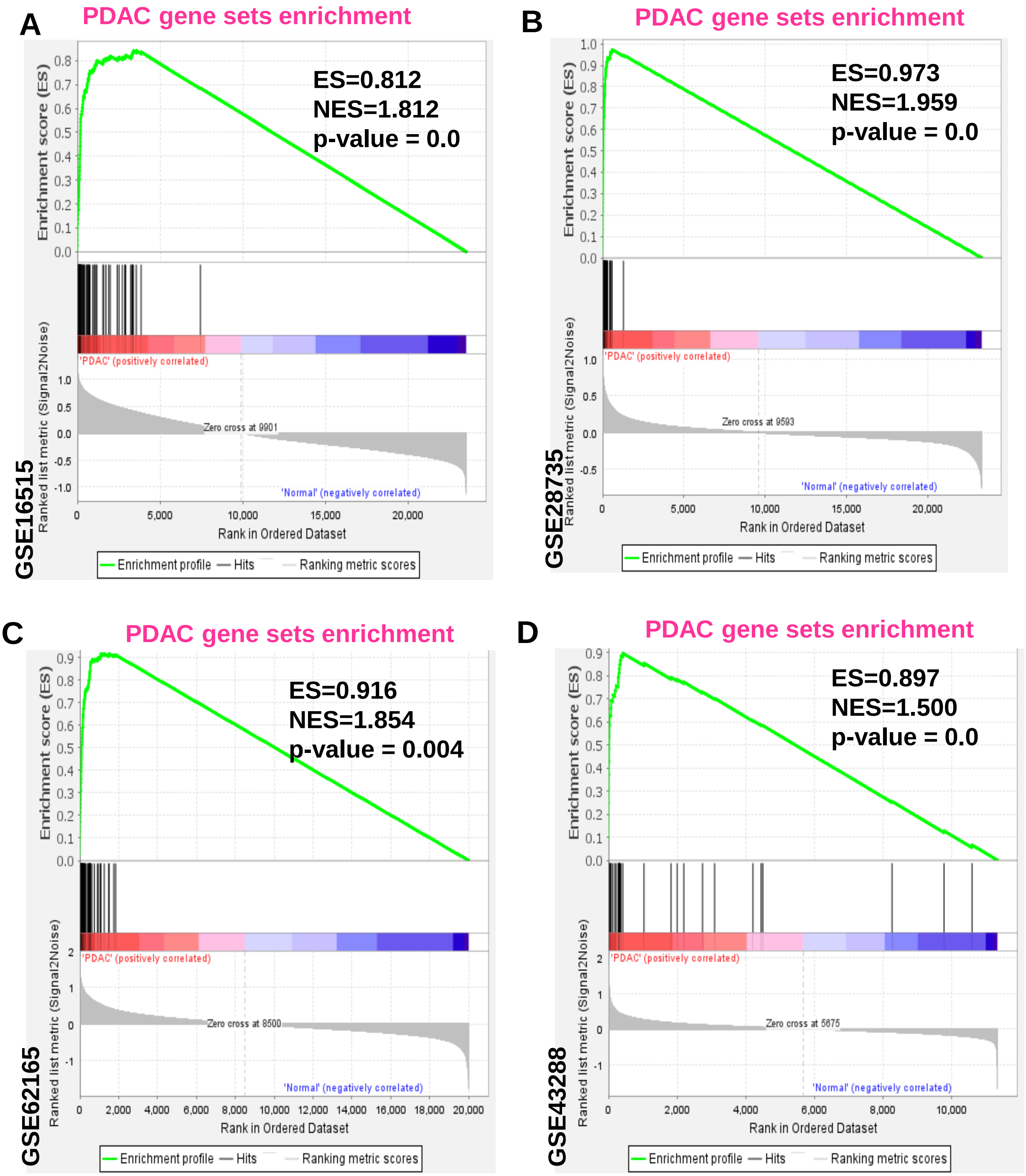


**Figure S2.** The PDAC hub genes were further confirmed with gene set enrichment analysis in those profiles. **A-D)** The results indicate greater and normalized enrichment scores with significant p values ($p<0.05$).

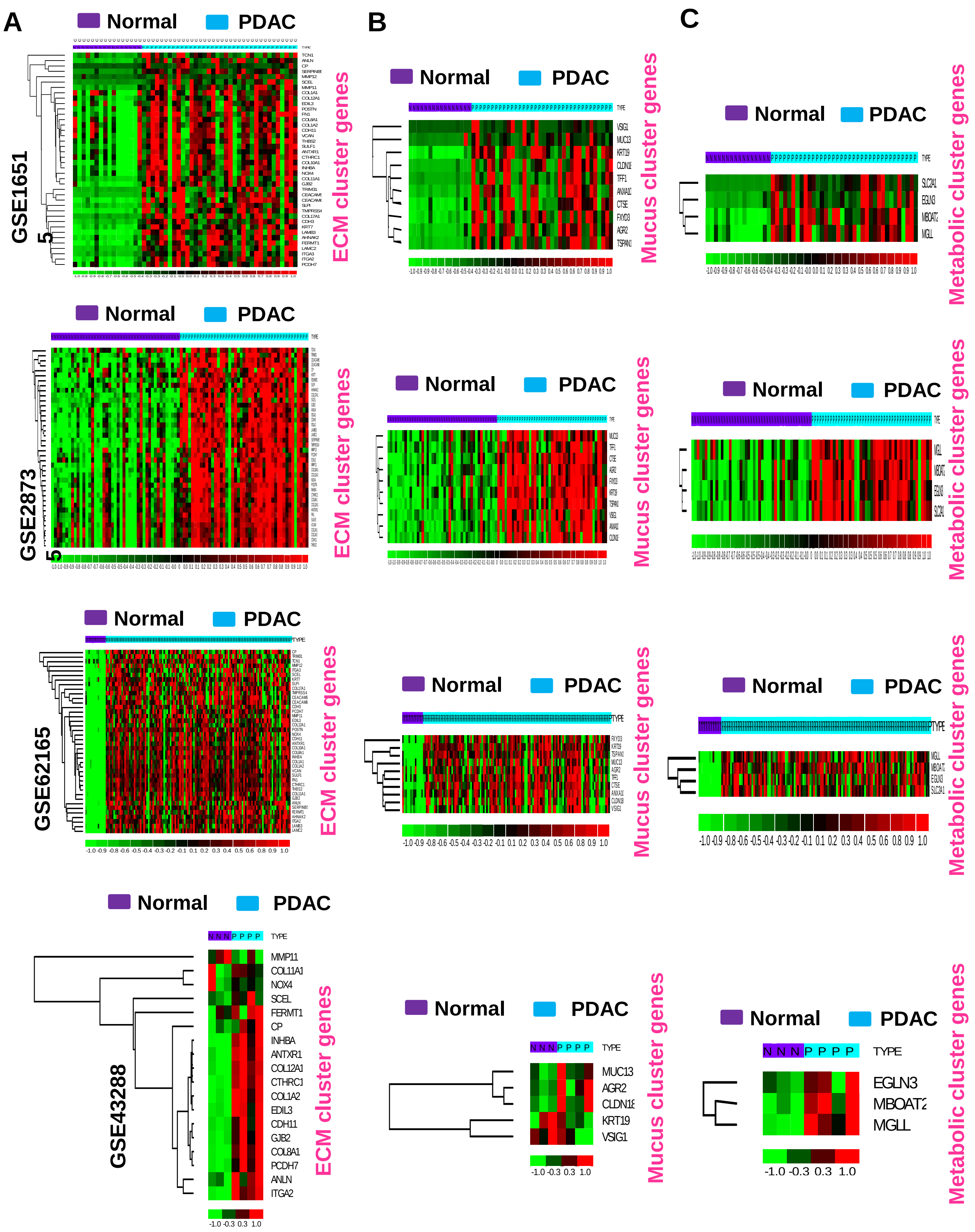


**Figure S3.** The expression patterns of the three cluster genes in four datasets. **A-C**) Each derived cluster is significantly enriched in PDAC tumors.

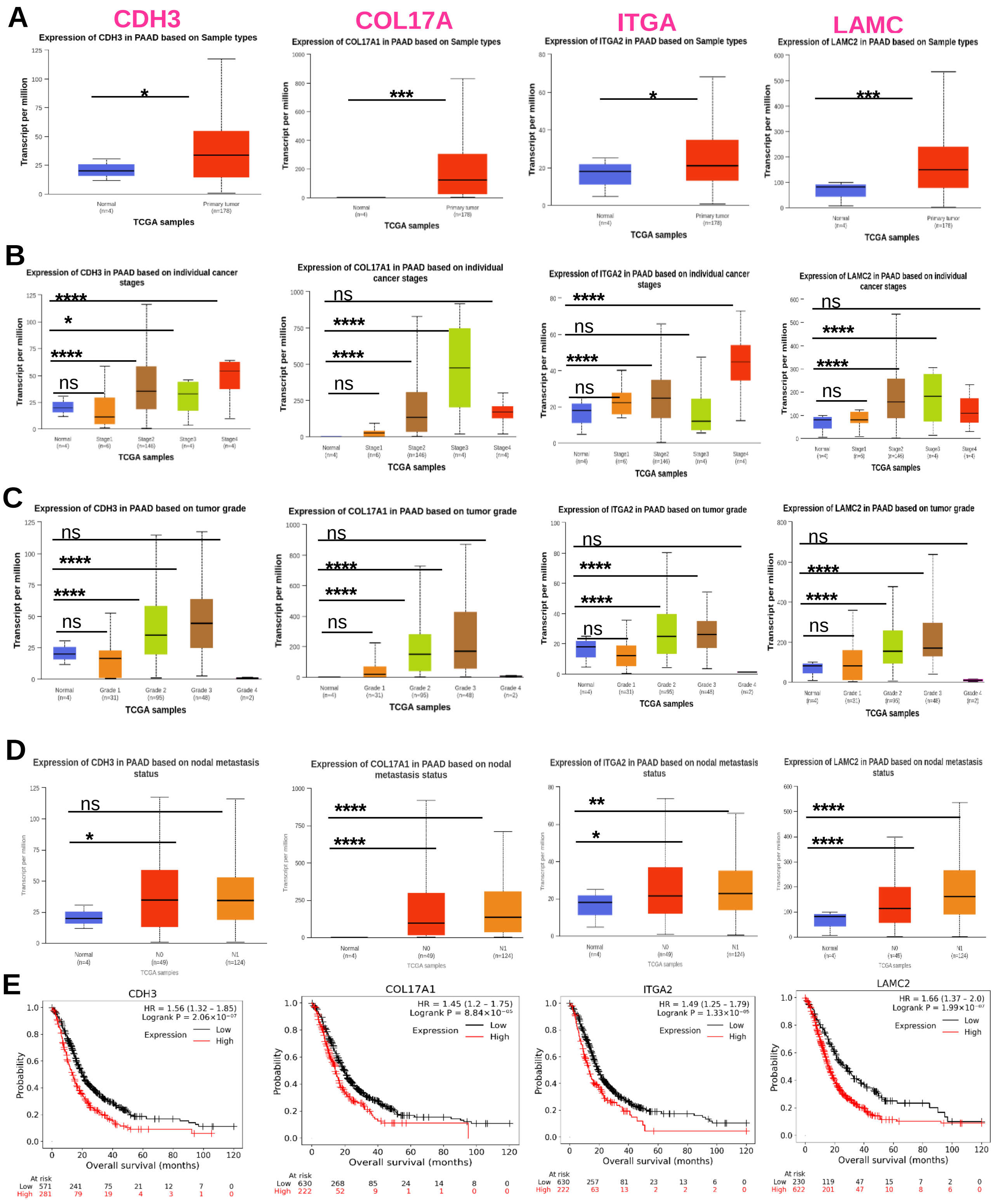


**Figure S4.** Validation of the ECM cluster genes' expression using TCGA dataset. **A-D**) The ECM genes' mRNA levels are elevated in primary tumors (A), late stage (B), high grade (C), and node N1 metastasis sample (D) compared with normal. **E)** High expression of ECM genes is corelated with poor survival in PDAC patients (****: p<0.0001, ***: p<0.001, **: p<0.01, and *: p<0.05).

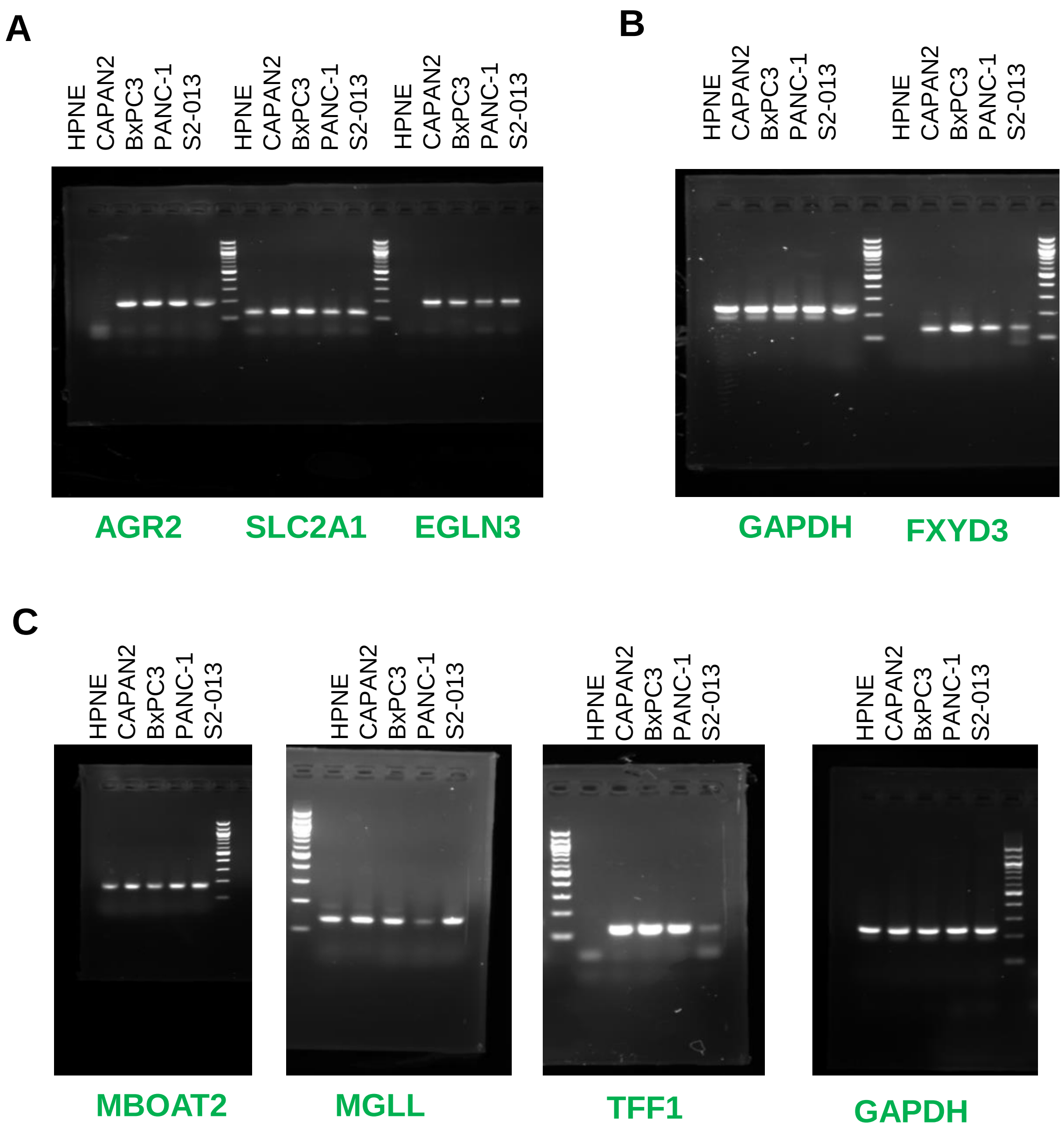


**Figure S5. A-C)** The uncropped original gel images for the validated genes of RT- PCR products.
